# Retrospective Reports of Weight Change and Inflammation in the US National Health and Nutrition Examination Survey

**DOI:** 10.1155/2013/601534

**Published:** 2013-02-11

**Authors:** Sarinnapha Vasunilashorn

**Affiliations:** Office of Population Research, Princeton University, 263 Wallace Hall, Princeton, NJ 08544, USA

## Abstract

*Purpose*. This study investigated the association between weight change and inflammation in a nationally representative population of US adults aged 40 and older. *Methods*. Using the US National Health and Nutrition Examination Survey (2005–2008), logistic regression models were used to determine the relationship between high levels of inflammation (C-reactive protein [CRP]) and infection (white blood cell count [WBC]) with 1- and 10-year change in self-reported weight status. *Results*. Change in 1- and 10-year weight was associated with high CRP but not high WBC. Individuals who gained or lost ≥10 kg had an odds of having high CRP that was 1.96 (95% CI 1.11–3.50) and 1.61 (95% CI 1.02–2.46) as high, respectively, as those who maintained a stable weight (<4 kg change) in the past year. The increased risk of elevated CRP among individuals who experienced at least 10 kg of weight loss or weight gain was also observed for weight change that occurred over the past 10 years; however, weight loss over the 10-year period was no longer associated with high inflammation. *Conclusions*. These results suggest that adult respondents who retrospectively self-reported weight loss or gain had higher levels of inflammation relative to their weight stable counterparts.

## 1. Introduction

It has been well established that obesity is associated with chronic low-grade inflammation [1, 2]. Far less is known, however, about the long-term effects of weight change on inflammation. Intentional weight loss has been shown, in the short term, to result in reduced inflammation [3, 4] but this relationship is not well understood in population-based samples. Using measured body mass index (BMI), retrospective studies of adults in the US, England, and France have suggested that weight gain is associated with increased systemic inflammation [5–8]. Several studies, however, do not have the ability to track changes in weight through measured anthropometry. As such, self-reported measures of current and past weight must be used. Little attention has focused on the relationship between self-reported measures of weight and indicators of health, such as blood levels of inflammation. One study reported that Japanese adults (age 40 and older) who self-reported a weight increase of 10 kg or more since age 20 had a higher mean CRP level than those whose weight remained stable [9].

It is unclear if the relationship between weight gain and inflammation is maintained across populations that vary by lifestyle-related behavioral and environmental factors. Interestingly, variations in body weight in Western and non-Western cultures have been recognized [10]; being thin is considered desirable in Western cultures, whiles plumpness is considered attractive in other societies (e.g., in China, fatness is associated with prosperity and longevity). The differences in cultural desirability surrounding body weight may result in variations in the relationships between body weight (and changes in body weight) and health-associated indicators. 

Although the relationship between retrospective reports of weight has been investigated in non-Western populations, this relationship has not been investigated in a nationally representative population of US adults. Given that variations in cultural desirability may result in differences in weight consciousness and the reporting of weight [11], examining the relationship between self-reported weight and inflammation among US adults represents an important step towards understanding the association between weight status and inflammation in a different social context. To further explore this relationship between self-reported weight change and inflammation and infection, the current study investigates the use of retrospective measures of self-reported weight and blood levels of C-reactive protein (CRP) and white blood cell count (WBC) in US adults. 

## 2. Methods and Procedures

### 2.1. Study Population

The current analysis was based on the US National Health and Nutrition Examination Survey (NHANES, 2005–2008). NHANES, conducted by the National Center for Health Statistics, regularly monitors the health and nutritional status of the US population [12]. Every year, approximately 5,000 individuals undergo detailed interviews and medical examinations, which include collection of several physiological measures. NHANES utilizes a complex sampling design but when weights are applied, the sample is representative of the noninstitutionalized American population. There were 2,708 individuals age 40 and older with information on sociodemographics, weight history, and blood inflammation and infection markers. The response rate for the NHANES 2005–2008 interview sample aged 40 and older was 71% and 69% for the study sample [13]. 

### 2.2. Measures

Participants were asked to report their current weight, weight one year ago, and weight 10 years ago. These retrospective, self-reports of weight were used to determine weight change relative to self-reported current weight. Weight change 1-year and 10-years prior was classified into five categories: loss ≥10 kg, loss 4–9 kg, stable (<4 kg change), gain 4–9 kg, and gain ≥10 kg [14]. Current BMI was calculated from self-reports of current weight (kg) divided by current height-squared (m^2^). 

Current levels of total cholesterol, CRP, and WBC were determined from whole blood. CRP was quantified using latex-enhanced nephelometry and analysis was conducted in the Department of Laboratory Medicine, Immunology Division at the University of Washington Medical Center. The lowest detectable concentration for CRP was 0.01 mg/dL, and the average interassay coefficient of variation (CV) was 5.1%. CRP provides a direct measure of serum protein that increases and decreases rapidly in response to acute inflammation or the destruction of tissue. WBC was determined by detecting and measuring changes in electrical resistance using the Coulter HMX Hematology Analyzer. The interassay CV for WBC was <2.5% with a pulse size wavelength of ≥35 fL. Sample-based quartiles of CRP and WBC were computed, and individuals in the highest quartile for each individual marker were labeled as having high CRP (>0.51 mg/dL) and high WBC (>8.1 × 1000 cells/*μ*L). 

### 2.3. Statistical Analysis

 Logistic regression models were used to evaluate associations between weight change (1-year and 10-year) and high CRP and high WBC. Model I adjusted for age (in years), sex, and current BMI. Model II adjusted for Model I covariates in addition to current smoking status (smoker versus nonsmoker) and total cholesterol levels. For all models, individuals who maintained a stable weight (<4 kg change) were the designated reference group. All analyses were performed using SAS 9.2 (SAS Institute, Inc., Cary, NC). The *surveylogistic* procedure and sample weights were used to account for the complex survey design. 

## 3. Results 

 Study characteristics of the participants have been summarized in Table 1. On average, individuals were approximately 60-years old (from the available adjudicated age; range: 40–85 [topcoded]), slightly less than half were male (47.59%), and 21.27% were current smokers. Mean CRP and WBC levels were 0.5 mg/L and 7500 cells/*μ*L respectively, and participants reported a current BMI of, on average, 28.51 kg/m^2^. Retrospective self-reports of weight change in the past year indicated that most individual's weight remained stable (63.91%), about 20% lost weight (13.81% lost 4–9 kg and 6.19% lost ≥10 kg), and slightly more than 16% gained weight (12.84% gained 4–9 kg and 3.25% gained ≥10 kg). The proportion of participants with stable weight 10 years prior (33.28%) was lower compared to that of weight reports 1 year prior. Weight gain in the past 10 years was prevalent in about half of the sample (25.65% gained 4–9 kg and 24.44% gained ≥10 kg), with far fewer individuals having lost weight during this time period (9.72% lost 4–9 kg and 6.91% lost ≥10 kg).

Relative to individuals whose weight remained stable, mean levels of CRP and WBC were higher among those who gained weight and, were slightly higher among those who lost weight (Table 2). CRP levels among individuals who gained ≥10 kg one year prior to the interview were, on average, 0.35 mg/dL higher compared to those whose weight was stable (<4 kg) 1 year prior, and 50% of those who gained ≥10 kg within the year had high CRP levels (i.e., were in the highest sample-based CRP quartile). Adults who lost ≥10 kg in the past year exhibited CRP levels that were, on average, 0.12 mg/dL higher than their stable weight counterparts. Weight gain in the past 10 years showed a similar relationship with CRP levels: mean CRP was 0.67 mg/dL for those who gained ≥10 kg, 0.42 mg/dL for those whose weight was stable (<4 kg change), and 0.54 mg/dL for individuals who lost ≥10 kg. The stable weight group also had the smallest percent of individuals with high CRP levels (15.86%) compared to 38.54% among the highest weight gaining group (≥10 kg) and 24.52% among the highest weight loss group (≥10 kg). 

 As with CRP levels, greater WBC was observed among individuals who gained and lost weight compared to those whose weight remained stable (Table 2). Within the one year interval, the average WBC for weight gainers (≥10 kg) was 7,460 cells/*μ*L, 7,110 cells/*μ*L for weight stable individuals, and 7,270 cells/*μ*L for those who lost ≥10 kg. High WBC was observed in 30.63% and 29.38% of people who reported losing or gaining ≥10 kg in the past 10 years, respectively.

 After adjusting for age, sex, and current BMI, participants who reported losing ≥10 kg had a higher odds of having elevated CRP relative to participants whose weight remained stable (<4 kg change) in the past year (odds ratios [OR] 1.67, 95% confidence interval (CI) 1.08–2.60; Table 3, Model I). Individuals who gained ≥10 kg in the past year had an odds that was 1.94 times that of participants with stable weight. A weight gain or loss of less than 10 kg in the past year was not significantly associated with elevated CRP levels. The addition of smoking status and total cholesterol levels (Model II) did not change the significance of the relationship between weight loss or gain of ≥10 kg and high CRP (weight loss: OR 1.61, 95% CI 1.03–2.50; weight gain: OR 1.96, 95% CI 1.10–3.50); the association between losing or gaining 4–9 kg in the past year and high CRP remained nonsignificant. 

When considering reports of weight change in the past 10 years, weight gain remained associated with elevated CRP levels while a lack of a relationship between weight loss and elevated CRP was observed. Individuals who gained weight in the past 10 years had greater odds of having high CRP compared to their stable weight counterparts (OR 1.62, 95% CI 1.16–2.27; Table 3, Model I), after adjusting for age, sex, and current BMI. Additionally controlling for current smoking status and total cholesterol did not attenuate the magnitude or alter the significance of the relationship between extreme weight gain within the past 10 years and high CRP (OR 1.62, 95% CI 1.16–2.26; Table 3, Model II). Lastly, no association between weight change and WBC in the 1- or 10-year interval was observed.

## 4. Discussion

This study finds that retrospective reports of change in weight over a one-year (and over an extended, 10-year) interval was associated with elevated CRP. In the fully adjusted models, adults who gained or lost ≥10 kg in the past year had higher odds of having elevated CRP compared to their stable weight counterparts. This relationship was also observed for individuals who gained ≥10 kg in the past 10 years relative to those whose weight remained stable. These findings correspond with previously reported studies of measured weight [5–8] and self-reported weight change [9]. 

 The relationship between weight gain and CRP reported in the current study aligns with prior research [5]; yet the mechanism that links weight status and CRP levels is not certain. Some have hypothesized that inflammatory factors chronically stimulate the sympathetic nervous system, which may in-turn result in weight increases. An alternate explanation for the association between weight gain and CRP is that inflammation is not directly associated with weight gain, but instead serves as a surrogate indicator of other processes related to weight gain, which were not measured in the current analysis. 

 The current study's finding of an increased odds of having elevated CRP among individuals who lost ≥10 kg may initially seem to counter previous reports that weight loss is associated with lower CRP levels. Such studies include participants who have intentionally lost weight (e.g., enrolled in weight programs or underwent lifestyle interventions) and those whose weight loss was unintentional. The unintentional weight losers likely reflect those who are older and have more medical conditions that may not be related to, or are indirectly associated with, weight changes. Older adults, who comprise a majority of our sample population, are believed to be more prone to weight loss than to weight gain. They also have additional comorbidities (e.g., cardiovascular disease), which have been associated with elevated CRP.

 The findings reported in the current study contribute to the existing literature by further detailing the relationship between weight change and blood inflammatory levels in a Western population. This is an important contribution to our understanding of weight status and health with respect to different societal and social contexts. Given that a similar relationship between self-reported weight change and CRP in a US (the current study) and Japanese population [9] were observed, this suggests that the relationship between self-reported body weight and inflammation are similar in populations with different social, cultural, and societal contexts.

 The lack of an association between weight change and WBC in the current study corresponds with previous reports in a rural Japanese population aged 40 and older [9]. However, an association was observed among Japanese office workers [15], who were younger than participants in the current study and in the study by [9]. This may be because, in some populations, older adults generally have lower WBC than younger adults [16] and that WBC may not be associated with weight change in late life. 

 The current study has a number of strengths. The retrospective reports of weight allowed for consideration of weight change that spanned 10 years, which is a modest interval of “observation”. This prohibits substantial dilution of the effect that inflammatory measures may have on weight change or that weight change may have on inflammatory measures. Causation, however, underscores a limitation of the current analysis. Causality cannot be inferred given several reasons: (1) CRP and WBC were measured at a single time point—it cannot be determined whether change in weight is associated with change in blood levels of these markers and (2) lack of ability to determine whether weight gains (or loses) results in increased (or decreased) CRP levels. Studies have suggested that inflammation may, in fact, predate weight gain [17]. The Atherosclerosis Risk in Communities (ARIC) Study and the Healthy Women Study, for example, reported high CRP concentrations prior to weight gain [16, 18]. An additional limitation is rooted in the use of self-reports of current and previous weight, which may not accurately or reliably be a proxy for measured weight. Bias and errors in reporting of weight have been shown to be greater in overweight females and males [19]. In the current study, no interactions were observed for sex by weight change (in the past 1-year or 10-years) or sex by current BMI. Use of recalled weight has been validated in epidemiological studies [20, 21], and an association between retrospective reports of weight change from age 20 and CRP levels have been observed in US women [16]. Lastly, we are unable to determine whether extreme weight loss was accompanied by a greater proportion of detrimental loss of lean mass or the beneficial loss of fat mass. Among adults who lost weight, it is expected that those who lost a greater proportion of lean body mass relative to fat mass would exhibit higher circulating levels of CRP. Our inability to distinguish between gains and losses of lean mass and fat mass may, in part, explain the lack of association observed between weight losses or gains of <10 kg and CRP levels. If gain in fat mass is largely what is driving the link between weight gain and inflammation, the current literature would benefit from attempts to disentangle differences in changes in lean body mass and fat mass with inflammation. 

 In conclusion, this study found that retrospective reports of weight gain were associated with CRP levels in a nationally representative population of US adults. Findings from the current study suggest that retrospective reports of weight may be an appropriate indicator of previous weight when anthropometric measures are not available. Future studies are required to determine the direction of this relationship and the mechanisms by which inflammation is associated with fluctuations in weight and the links between change in lean body mass, fat mass, and inflammation.

## Figures and Tables

**Table 1:** Characteristics of persons age 40 and older in the US NHANES 2005–2008 (*n* = 2,708).

	Mean ± SD or %
Age (years)*	60.21 ± 13.52
Men (%)	47.59
Current smoker (%)	21.27
Inflammation and infection	
C-reactive protein, mg/L*	0.5 ± 0.8
White blood cell count, ×1000 cells/*μ*L*	7.5 ± 2.6
Weight status	
Body mass index (kg/m^2^)*	28.51 ± 6.27
Weight change in past 1 year (%)	
Loss	
≥10 kg	6.19
4–9 kg	13.81
Stable (<4 kg)	63.91
Gain	
4–9 kg	12.84
≥10 kg	3.25
Weight change in past 10 years (%)	
Loss	
≥10 kg	6.91
4–9 kg	9.72
Stable (<4 kg)	33.28
Gain	
4–9 kg	25.65
≥10 kg	24.44

*Unweighted averages were reported in order to obtain standard deviations (SD).

**Table 2:** Mean levels and percentages of high levels* of C-reactive protein and White blood cell count by 1- and 10-year change in weight categories.

	C-reactive protein (mg/dL)			White blood cell count (×1000 cells/*μ*L)		
	*N*	Mean ± SD	% High	*N*	Mean ± SD	% High
Weight change in past 1 years (%)						
Loss						
≥10 kg	96	0.59 ± 0.74	38.82	96	7.27 ± 2.11	32.90
4–9 kg	323	0.43 ± 0.64	22.48	323	7.29 ± 3.09	29.31
Stable (<4 kg)	1721	0.47 ± 0.93	20.47	1716	7.11 ± 2.53	24.84
Gain						
4–9 kg	378	0.59 ± 0.70	30.86	378	7.36 ± 2.15	31.87
≥10 kg	177	0.82 ± 0.98	50.18	177	7.46 ± 2.19	30.85
Weight change in past 10 years (%)						
Loss						
≥10 kg	220	0.54 ± 0.88	24.52	220	7.15 ± 2.21	30.63
4–9 kg	297	0.40 ± 0.65	20.25	295	7.03 ± 1.88	26.77
Stable (<4 kg)	881	0.42 ± 8.88	15.86	878	7.07 ± 2.35	23.66
Gain						
4–9 kg	626	0.47 ± 0.92	22.16	626	7.22 ± 3.35	28.53
≥10 kg	642	0.67 ± 0.87	38.54	642	7.41 ± 2.24	29.38

*High levels included individuals in the highest sample-based quartile (CRP >0.51 mg/dL; WBC > 8.1 × 1000 cells/*μ*L).

SD: standard deviation.

**Table 3:** Odds ratios from logistic regression models predicting high levels of C-reactive protein and White blood cell count by 1- and 10-year change in weight categorizes.

	High C-reactive protein*				High White blood cell count*			
	Model I		Model II		Model I		Model II	
	OR	(95% CI)	OR	(95% CI)	OR	(95% CI)	OR	(95% CI)
Weight change in past 1 year	*n* = 2685				*n* = 2681			
Loss								
≥10 kg	**1.67**	**(1.08–2.60)**	**1.61**	**(1.03**–**2.50)**	1.25	(0.81–1.93)	1.13	(0.73–1.75)
4–9 kg	0.98	(0.70–1.37)	0.92	(0.70–1.38)	1.21	(0.89–1.64)	1.22	(0.89–1.67)
Stable (<4 kg)	Reference		Reference		Reference		Reference	
Gain								
4–9 kg	1.28	(0.70–1.37)	1.30	(0.92–1.83)	1.22	(0.89–1.68)	1.27	(0.91–1.77)
≥10 kg	**1.94**	**(1.10–3.43)**	**1.96**	**(1.10–3.50)**	0.97	(0.54–1.74)	0.97	(0.55–1.73)

Weight change in past 10 years	*n* = 2657				*n* = 2653			
Loss								
≥10 kg	1.24	(0.78–1.99)	1.24	(0.77–2.00)	1.21	(0.78–1.86)	1.19	(0.77–1.84)
4–9 kg	1.26	(0.83–1.90)	1.23	(0.82–1.86)	1.29	(0.88–1.90)	1.21	(0.82–1.78)
Stable (<4 kg)	Reference		Reference		Reference		Reference	
Gain								
4–9 kg	1.24	(0.91–1.69)	1.27	(0.93–1.73)	1.01	(0.75–1.36)	1.06	(0.78–1.42)
≥10 kg	**1.62**	**(1.16**–**2.27)**	**1.62**	**(1.16**–**2.26)**	0.96	(0.69–1.33)	0.92	(0.66–1.29)

*High levels included individuals in the highest sample-based quartile (CRP > 0.51 mg/dL; WBC > 8.1 × 1000 cells/*μ*L).

Model I adjusts for age, sex, and current BMI.

Model II adjusts for current smoking status and total cholesterol in addition to Model I covariates.

OR: odds ratio; CI: confidence interval.

Bold indicates significant at the .05 level.
